# Effects of precipitates and dislocation loops on the yield stress of irradiated iron

**DOI:** 10.1038/s41598-018-25285-z

**Published:** 2018-05-02

**Authors:** Arttu Lehtinen, Lasse Laurson, Fredric Granberg, Kai Nordlund, Mikko J. Alava

**Affiliations:** 1 Department of Applied Physics, COMP Centre of Excellence, Aalto University, Aalto, Espoo, P.O. Box 11100, FIN-00076 Finland; 2 Department of Physics, University of Helsinki, Helsinki, P.O. Box 43, FIN-00014 Finland

## Abstract

Plastic deformation of crystalline materials is governed by the features of stress-driven motion of dislocations. In the case of irradiated steels subject to applied stresses, small dislocation loops as well as precipitates are known to interfere with the dislocation motion, leading to an increased yield stress as compared to pure crystals. We study the combined effect of precipitates and interstitial glissile $\frac{{\bf{1}}}{{\bf{2}}}\langle {\bf{111}}\rangle$ dislocation loops on the yield stress of iron, using large-scale three-dimensional discrete dislocation dynamics simulations. Precipitates are included in the simulations using our recent multi-scale implementation [A. Lehtinen *et al*., Phys. Rev. E 93 (2016) 013309], where the strengths and pinning mechanisms of the precipitates are determined from molecular dynamics simulations. In the simulations we observe dislocations overcoming precipitates with an atypical Orowan mechanism which results from pencil-glide of screw segments in iron. Even if the interaction mechanisms with dislocations are quite different, our results suggest that in relative terms, precipitates and loops of similar sizes contribute equally to the yield stress in multi-slip conditions.

## Introduction

It is well established that plastic deformation of pure crystals is mainly due to the stress-driven motion of dislocation lines. In many practical settings, however, dislocations are not the only defect type present within the crystals. Experimental studies on pristine and irradiated metals have shown complex micro- and nanostructure which in addition to dislocations include voids^1–3^, bubbles^4^, interstitial clusters^3,5^, precipitates^4,6–10^ and dislocation loops^1,2,4,11^.

In general, these different defects serve as obstacles for dislocation motion, and consequently mechanical tests on irradiated steel samples have shown that irradiation will increase the yield strength, but at the same time also the yield drop becomes larger in magnitude^5,12^. The ductile-brittle transition temperature has also been showed to increase with radiation dose, which in some cases can become close to the operating temperatures of nuclear power plants^13–15^. These examples demonstrate the importance of understanding the role of irradiation-induced nanoscale features on the macroscopic behavior of steels which are important materials in constructing structures in highly irradiated environments.

The effect of these different features on the dynamics of individual dislocation lines has been studied in detail with molecular dynamics (MD) simulations for some time. Recent examples include refs^16–24^. MD simulations give valuable insight into the physical mechanisms of dislocation-defect interactions, but the computational limitations of simulating materials at atomic resolution constrain the system size usually to a singular dislocation and a few defects. For the purpose of simulating the collective behavior of several dislocations interacting with a large number of obstacles, coarse-grained, mesoscale descriptions are necessary. To this end, we consider here large-scale three-dimensional (3D) discrete dislocation dynamics (DDD) simulations. In such DDD simulations, the basic degrees of freedom of the systems are taken to be the dislocation lines, and the atomic scale details of the embedding crystal lattice enter only via various constraints on the motion of the dislocations. The curved dislocation lines are represented by straight line segments whose interactions are obtained from linear elasticity theory. This approximation makes it possible to simulate 3D crystals of linear sizes up to several micrometers, containing a large number of dislocations.

Previous DDD studies addressing the effect of strong immobile obstacles on dislocation motion, and thus on the deformation process, have focused on the interaction of dislocations with different precipitates in single slip^25–27^ and multiple slip systems^28^. These simulations show dislocations bypassing these strong obstacles with the standard Orowan looping mechanism. In FCC metals more complex obstacles like irradiation-generated stacking-fault tetrahedra and immobile Frank loops have been studied^29^. These serve as pinning points for straight dislocations but dislocations can also absorb them in some cases leading to obstacle-free channels where the plastic deformation localizes.

In the case of BCC iron, irradiation generates mixed population of self-interstitial atom (SIA) prismatic dislocation loops consisting of 〈100〉 and $\frac{1}{2}\langle 111\rangle$ types^30–32^. Because of their dislocation nature that generates an anisotropic stress field^33^, SIA loops interact with straight dislocations in a complicated manner. The strength of interaction depends on their Burgers vectors and orientations. Previous DDD simulations of BCC iron containing these kind of loops and dislocations have been able to capture the increase in the yield stress and the generation of obstacle free-channels^34^. The channels are formed when line dislocations push and destroy the loops. Recently Cui *et al*. simulated compression of irradiated Fe micropillars^35^. Their DDD model included loop absorption and other mechanism but they did not observe channel formation. They argued that this was due to the low loop density in their simulations, which indeed was lower than that of Arsenlis *et al*.^34^.

Simulations with both prismatic loops and strong precipitates have been performed in single slip systems^36^. In most real crystals multiple slip systems are simultaneously active, with dislocations forming complex 3D networks, involving also dislocation junctions, serving as sources for dislocation multiplication^37^, and consequently contributing to an increase of the dislocation density with the accumulated plastic strain. In order to realistically model the interplay of such complex multi-slip dislocation dynamics and the collective effects of both irradiation-induced defects such as mobile prismatic dislocation loops and immobile precipitates interacting with the dislocations, full 3D DDD simulations with various obstacle types incorporated within the simulations are needed.

In this work we study the effect of immobile precipitates and mobile SIA prismatic dislocation loops of $\frac{1}{2}\langle 111\rangle$ nature, as well as that of various mixtures of the two, on the yield stress of iron crystals at high temperatures, using our recently developed multi-scale approach to model precipitates within DDD simulations^38^. By combining atomistic simulations with DDD we can simulate collective dislocation processes with physically justifiable parameters and thus obtain more accurate knowledge of the mechanical properties of irradiated steels at the micron scale. The main result is that precipitates and loops of similar sizes are effectively equally strong obstacles for dislocation motion in multi-slip conditions, and their contribution to the the yield stress is of similar magnitude.

## Results

### Simulation setup

The DDD simulations are performed using a modified version of the DDD code ParaDiS^39^, where we have added a new obstacle data structure which is used to model immobile spherical defects like precipitates^38^, see Methods for details. Our interest is in the yielding process of BCC iron at elevated temperatures, and for this reason dislocation mobility parameters and the elastic constants were taken from MD simulations at the temperature of 750 K, i.e., close to the typical operating temperatures of nuclear reactors. At these temperatures it has been observed that screw dislocations in BCC lattice can move in non-crystallographic glide planes because the Peierls landscape of their core changes as a function of stress^40^; this phenomenon is known as pencil glide^41^.

To include this feature in our simulations we use the mobility function BCC_0 which is distributed with the default version of ParaDiS. The chosen mobility function models pencil glide by assuming glide-constrained linear mobility for dislocation segments with edge character and isotropic linear mobility for dislocation segments with screw character^39,42^. Linearity of the mobility function was justified by the MD simulations which showed a linear relation with the dislocation velocity as a function of the applied stress at 750 K^38^.

The simulation box was chosen to be a cube with the dimensions of 0.75 *μ*m×0.75 *μ*m×0.75 *μ*m with periodic boundary conditions in all directions in order to model bulk properties of the crystal in the sense that there are no effects related to the presence of free surfaces. The initial configuration of dislocations consisted of 24 straight screw dislocations in the $\frac{1}{2}\langle 111\rangle \{110\}$ slip system, which corresponds to an initial dislocation density of ${\rho }_{0}=7.38\times {10}^{13}\,{{\rm{m}}}^{-2}$. In addition to straight dislocations, varied amounts of precipitates and prismatic dislocation loops were randomly positioned within the simulation box. To prevent overlapping, a minimum distance between precipitates was set to match their size parameter. The precipitate strength parameter was chosen to be $A=1.56\times {10}^{-18}\,\,{\rm{Pa}}\,{{\rm{m}}}^{3}$, which, when considering the size parameters *R*_*p*_ = 5 and 10 nm, is large enough to make the precipitates strong (Orowan loop generating) obstacles for dislocations moving within a plane spanned by the dislocation line and the precipitate center^38^. The precipitate number density varied in the range ${\rho }_{{\rm{p}}}=1.0\times {10}^{21}\cdots 2.0\times {10}^{22}\,{{\rm{m}}}^{-3}$, which is within the range of typical experimental values for oxide dispersion strengthened steels^10^. Dislocation loops were perfect interstitial hexagonal loops of $\frac{1}{2}\langle 111\rangle$ nature. These kinds of loops have been observed experimentally in irradiated iron^11,30,31,43,44^.

Strain-rate controlled load is implemented in ParaDiS by changing the stress in every time step according to the equation $d\sigma =G\cdot (\dot{\varepsilon }\cdot dt-d{\varepsilon }_{plastic})$, with *G* being the shear modulus. Thus, the simulated crystal has its own inherent elasticity but the machine stiffness is infinite which is similar to the extreme strain control case studied by Cui *et al*.^45^. Using a softer machine would lead to a decreased damping of the temporal fluctuations in the deformation process. However, the limit of a stiff machine we consider here is a reasonable approximation of a typical deformation experiment where the element applying the deformation is stiffer than the sample being deformed. Unless stated otherwise, all systems were strained with a constant strain rate of $\dot{\varepsilon }=1\times {10}^{6}\,{{\rm{s}}}^{-1}$ in the [001] direction. Relevant simulation parameters are presented in Table 1.

**Table 1:** Simulation parameters.

Parameter	Value
*L*_box_	0.75 *μ*m
*ρ*_0_	7.38 × 10^13^ m^−2^
*b*	0.2502 nm
*r*_core_	2.9*b*
*E*_core_	$1.84\,\frac{{\rm{eV}}}{b}$
*G*	75 GPa
*ν*	0.379
ParaDiS mobility function	BCC_0
*M*_edge_	6036.0(Pas)^−1^
*M*_screw_	6036.0(Pas)^−1^

### Effect of hard precipitates

**Figure 1:**
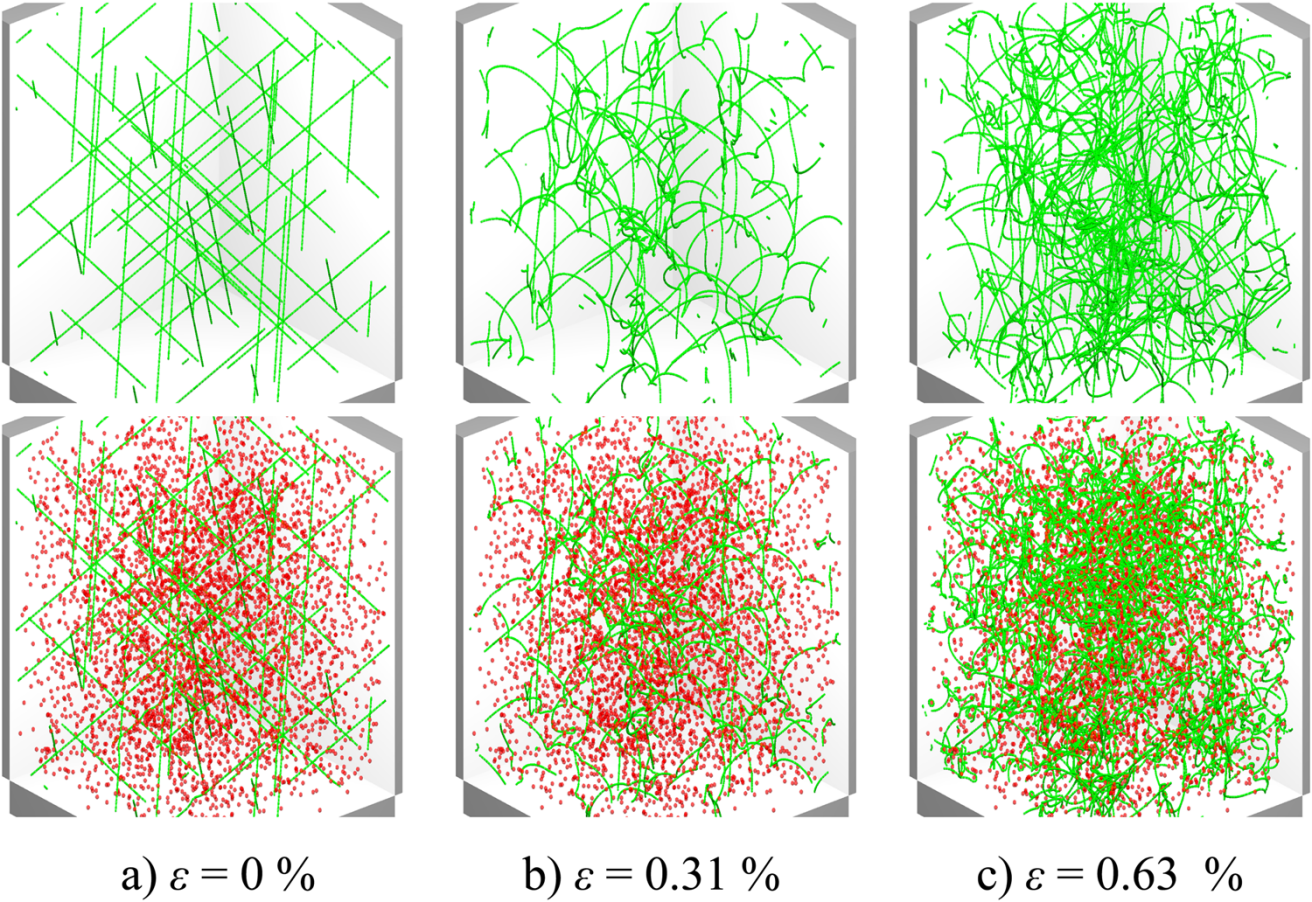
Snapshots of the initial and strained states of a dislocation system without precipitates (top) and of a system with hard precipitates (bottom). Precipitate density is *ρ*_*p*_ = 5.0 × 10^21^ m^−3^, and the size parameter of the precipitates is *R*_*p*_ = 5 nm.

First, we describe a simulation of a system containing initially straight dislocations without other defects. A strain rate of $\dot{\varepsilon }=1\times {10}^{6}\,{{\rm{s}}}^{-1}$ is imposed, and dislocations begin to move on their respective slip planes until they collide, see the top panel of Fig. 1a. During the early stages, deformation is mostly elastic as the movement of dislocations does not generate enough plastic strain to satisfy the imposed strain rate. Junctions are formed in the collisions which serve as pinning points for dislocation segments, see top panel of Fig. 1b. Under increasing stress the dislocations start to bow out from their pinned positions, i.e., they act as dislocation sources. This leads to an increase of the dislocation density and increased plastic strain until the crystal yields at the strain of *ε* ≈ 0.39%, see top panel Fig. 1c. Video of an example simulation is provided as Supplementary Material (Video 1).

**Figure 2:**
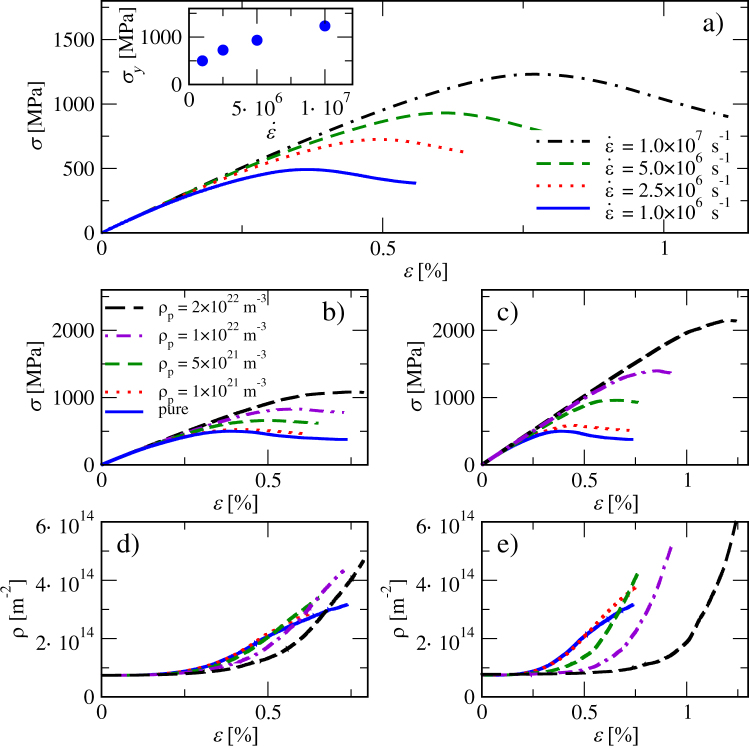
(**a**) Stress-strain curves of pure systems strained with different strain rates $\dot{\varepsilon }$. In the inset the yield stress *σ*_*y*_ is plotted as a function of $\dot{\varepsilon }$. (**b**) Shows stress-strain curves for different precipitate densities *ρ*_p_, considering precipitates with the size parameter *R*_p_ = 5 nm. (**c**) Shows the corresponding stress-strain curves for *R*_p_ = 10 nm. The evolution of the dislocation density *ρ* with strain for the same *ρ*_p_-values considered above, with *R*_p_ = 5 nm in (**d**) and *R*_p_ = 10 nm in (**e**), respectively.

The realistic dislocation mobilities obtained from MD simulations reduced the time-step in the DDD simulations. Because of this, the strain rate was set to a quite high value in order to get enough strain within a practical computation time. It is prudent to check if there are rate effects related to this. Hence, we performed simulations with different strain rates for the pure dislocation system. Stress-strain curves from these simulations are plotted in Fig. 2a). From the curves we measure the yield stress as the local maximum *σ*_*y*_(*ε*_*y*_) = max(*σ*(*ε*)). A clear rate effect, where the yield stress is proportional to the strain rate, is observed [inset of Fig. 2a)]. One may compare the resulting *σ*_*y*_ for the low strain rate limit to micropillar compression experiments^46^. In these experiments the yield stress showed significant stochastic variation and the average of five pillars was *σ*_*y*_ = 260 MPa. The largest measured value for single pillar was *σ*_*y*_ = 432 MPa which is of the same order as the simulated yield stress of *σ*_*y*_ = 500 MPa at $\dot{\varepsilon }=1\times {10}^{6}\,{{\rm{s}}}^{-1}$^46^. In what follows, all simulations are performed with $\dot{\varepsilon }=1\times {10}^{6}\,{{\rm{s}}}^{-1}$, a choice which may results in small deviations from the quasistatic limit.

Simulations with immobile precipitates and straight dislocations progressed in a similar manner as in the case of the pure dislocation system (bottom row of Fig. 1), the difference being that now immobile precipitates serve as obstacles for dislocation motion. In the stress-strain curves this is visible as an extended linearly elastic section at the beginning of the loading before yielding, as can be seen in (Fig. 2b and c). With increasing stress, dislocations bypass the precipitates by bowing around them and forming Orowan loops. Roughening of the dislocation lines leads to an increasing total dislocation length and thus to a higher dislocation density. In (Fig. 2d and e) dislocation density *ρ* is plotted as a function of *ε* for two precipitate sizes. The rate at which dislocation density increases is proportional to the number of precipitates in both cases. A Video of an example simulation is included as Supplementary Material (Video 2).

**Figure 3:**
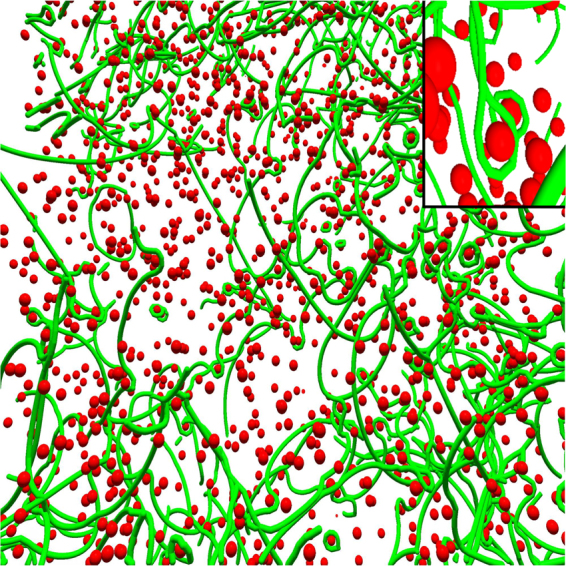
Close-up look at the 3D structure of a dislocation system with strong precipitates at a strain of *ε* = 0.63%. Dislocations (green lines) evolve into a complex network where they pin to each other forming junctions. Dislocations that have bypassed precipitates (red spheres) have left Orowan loops around some of the obstacles. In the inset there is an example of a twisted noose where the edge arms of the loops are on different glide planes and thus cannot annihilate each other.

A close-up of a strained microstructure is presented in Fig. 3. Dislocations that have moved over precipitates have left Orowan loops in their wake. Sometimes dislocations do not bypass the obstacles with the classical Orowan mechanism, but they stay pinned to the obstacle and form a twisted noose around it. There, the edge segments are on different glide planes, and hence cannot annihilate each other. An example can be seen in the upper right corner of Fig. 3.

The stress-strain curves show that the material strengthens proportionally to the precipitate density. To quantify this effect, we measure the yield stress and compare it to an analytical prediction. This prediction is obtained by the following analysis. Tensile straining generates a resolved shear stress on the glide plane of the dislocations pushing them over the obstacles, which in the pure system are forest dislocations. In precipitated system both forest dislocations and precipitates act as obstacles for the dislocation motion. The resolved shear stress is related to the tensile stress by a Schmidt factor *α*, *σ*_*r*_ = *ασ*_*y*_. Straight dislocations in our simulations were on the $\frac{1}{2}\langle 111\rangle \{110\}$ slip system for which the Schmidt factor is *α* = 0.4082. The linear rule of mixture *σ*_*tot*_ = (*σ*^pure^ + *σ*^precip^) is expected to be suitable for systems which contain few strong obstacles together with many weak obstacles^47^. This applies to our system as even though all of the precipitates are strong obstacles if dislocations interact with them at the equator plane, this is not true in the general case as dislocations can interact with the precipitates at arbitrary latitudes. By assuming the linear rule of mixture, the tensile yield stress of a precipitate-dislocation system can then be written as

1 $${\sigma }_{y}=\frac{1}{\alpha }({\sigma }_{r}^{{\rm{pure}}}+{\sigma }_{r}^{{\rm{precip}}})={\sigma }_{y}^{{\rm{pure}}}+\frac{1}{\alpha }{\sigma }_{r}^{{\rm{precip}}}$$

In order to evaluate the resolved shear stress ${\sigma }_{r}^{{\rm{precip}}}$ needed to overcome the precipitates we use the analytical expression obtained by Bacon *et al*.^48^. They considered a case of single dislocation driven quasistatically towards a row of impenetrable obstacles with a uniform size. Including dislocation self-interaction and effects from defect size they obtained an expression for ${\sigma }_{r}^{{\rm{precip}}}$ in this simple scenario. Based on these results and using a line tension approximation they proposed a more general formula for randomly positioned obstacles,

2 $${\sigma }_{r}^{precip}=C\frac{Gb}{L-D}{[\mathrm{ln}(\frac{L}{{r}_{{\rm{core}}}})]}^{-\frac{1}{2}}{[\mathrm{ln}(\frac{\bar{D}}{{r}_{{\rm{core}}}})+0.7]}^{\frac{3}{2}},$$

where $C=\frac{1}{2\pi }$, *L* is the distance between obstacles in the glide plane, *D* is the diameter of the obstacles, $\bar{D}=\frac{DL}{D+L}$ is the harmonic average of *L* and *D*, *G* is the shear modulus, *r*_core_ the dislocation core radius and *b* the Burgers vector magnitude. We denote this as the Bacon-Kocks-Scattergood (BKS) equation. However, we must perform some modifications because in our simulations precipitates are randomly distributed within the volume. Thus we must relate the inter-precipitate distance in the glide plane to the number density in three dimensions. For spherical obstacles the distance between them as a function of their number density is *L* = (2*Dρ*_*p*_)^−0.5^ ^49,50^. Thus, we modify Eq. (2) by adding the inter-particle distance for 3D distributed obstacles, and obtain for the yield stress

3 $${\sigma }_{y}={\sigma }_{y}^{pure}+\frac{Gb}{\alpha [{(2D{\rho }_{p})}^{-0.5}-D]}{[\mathrm{ln}(\frac{{(2D{\rho }_{p})}^{-0.5}}{{r}_{{\rm{core}}}})]}^{-\frac{1}{2}}{[\mathrm{ln}(\frac{\bar{D}}{{r}_{{\rm{core}}}})+0.7]}^{\frac{3}{2}}.$$

**Figure 4:**
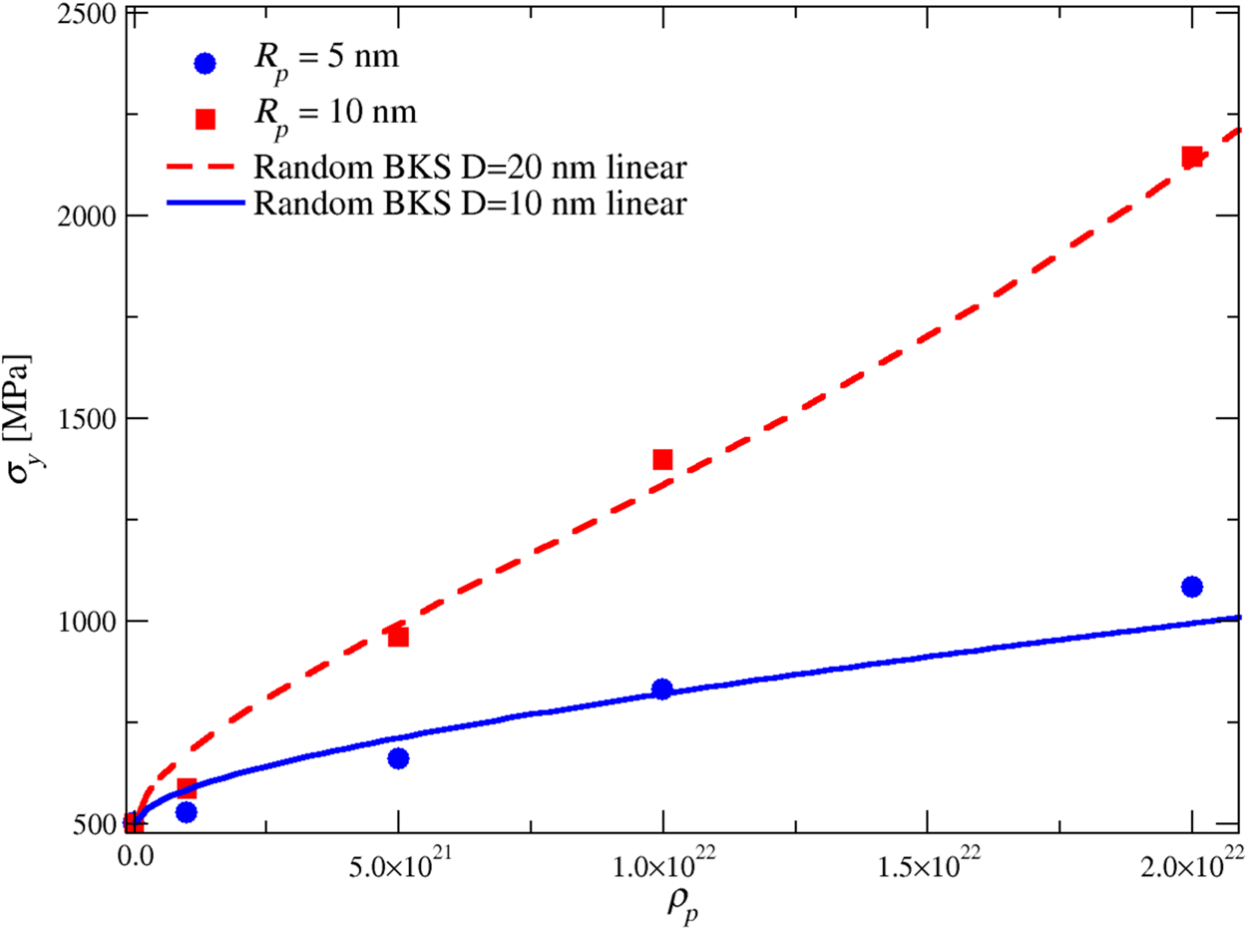
Yield stress as a function of precipitate density from simulations (symbols) and from the modified Bacon-Kocks-Scattergood (BKS) equation [Eq. (3), lines].

Examples of the yield stresses calculated from Eq. (3) are plotted in Fig. 4 together with yield stresses obtained from the simulations for two precipitate sizes.

For the smaller precipitate size we can observe that when *ρ*_*p*_ < 1 × 10^22^, the yield stresses from Eq. (3) (blue line) are larger than the simulated ones (blue circles). There are a number of reasons that can be behind this difference. The glide planes of dislocations cut the precipitates randomly in 3D, so the dislocations do not see a uniformly sized precipitate population but a distribution of precipitates with different diameters. The maximum diameter in this distribution is the equator diameter, while the minimum is arbitrarily small. When applying Eq. (3), we assumed that all obstacles have the same diameter, the equator one. It is easier for dislocation to bypass arbitrarily small obstacles so this could be one reason why the simulations with smaller precipitate densities give smaller yield stress values than those obtained from BKS. The formula was obtained by considering only the dislocation self-interaction in the case where the dislocation is curved around an infinitely hard exactly spherical obstacle which is positioned randomly on the glide plane. This approximation differs from our DDD implementation where there is a continuous stress-field around the obstacles: the effective size of the particles could also be larger than the size parameter would suggest. This could explain why the yield stresses for larger precipitates (red squares) are a bit higher than predicted by the modified BKS equation (red dotted line). Relatively small changes in obstacle size, say, from *D* = 20 nm to *D* = 30 nm, will increase the yield stress predicted by the BKS model significantly especially at higher obstacle densities. When the precipitate density is large, i.e., *ρ*_*p*_ > 1 × 10^22^, the dislocation density also increases, leading to stronger forest hardening. In addition the screw dislocations can move in non-crystallographic directions which is not taken into account in the BKS equation. Lastly it is important to notice that the BKS formula is obtained by considering quasistatic loading conditions, while in our simulation we use a relatively high strain rate which can lead to overestimation of the yield stress.

### Relative effect of precipitates and dislocation loops

Arsenlis *et al*. simulated several elementary reactions between straight dislocations and prismatic loops^34^. According to their results loops can be repelled by line dislocations but also attracted to them depending on the Burgers vectors and orientations of the loops and line dislocations.

If the loops and the line dislocation have the same Burgers vector and the line is a screw there is no elastic interaction. The loop is absorbed to the dislocation as a helical turn (see Video 3, showing the process as observed in our simulations considering a strain rate of $\dot{\varepsilon }=1\times {10}^{6}\,{{\rm{s}}}^{-1}$). If the Burgers vectors differ the loops act as strong pinning points. An edge dislocation with the same Burgers vector as the loop has an elastic interaction with it. The dislocations push or drag the loops depending on their relative orientation, but the loop does not pin the dislocation. If the Burgers vectors are different for the loop and the line dislocation there is always an elastic interaction. Depending on the orientation, loops are repelled by the line dislocation or are attracted towards it. In the latter case, if the Burgers vectors of the loops are on the glide plane of the line dislocation, the loops do not impede the dislocation motion but are dragged along with them. If the loop’s Burgers vector is not in the line dislocation’s glide plane, the loop acts as a pinning center for the dislocation line.

**Figure 5:**
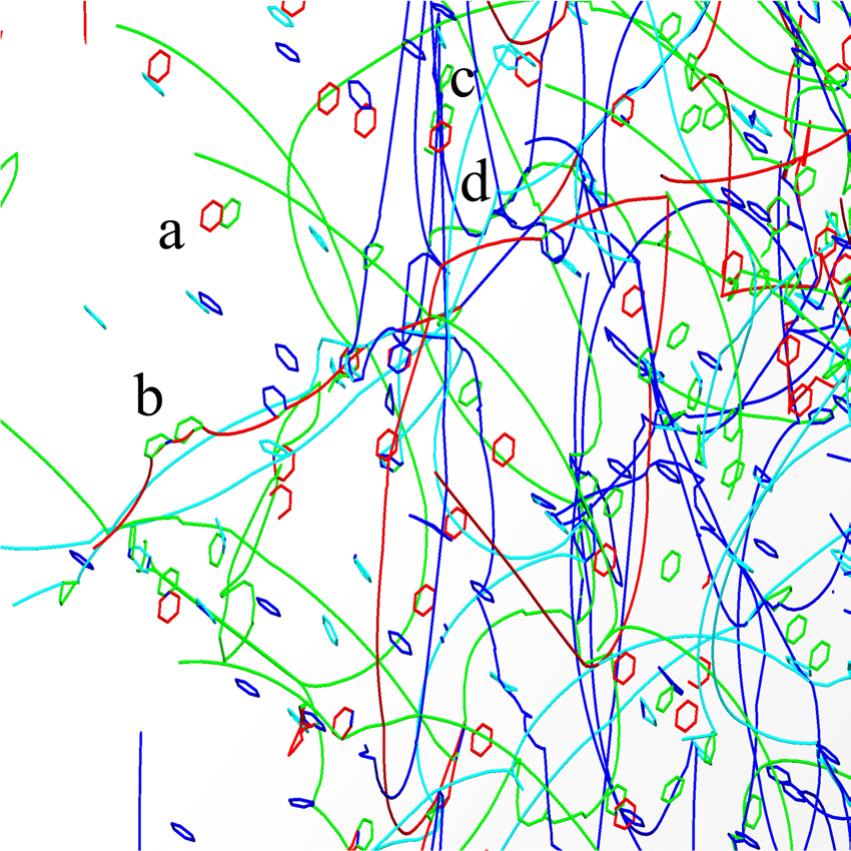
Close-up view of the 3D structure of a dislocation system with glissile dislocation loops at a strain of *ε* = 0.5%. Different Burgers vectors are indicated with different colors as follows: dark blue $b=\frac{1}{2}\langle 111\rangle$, cyan $b=\frac{1}{2}\langle 11\bar{1}\rangle$, green $b=\frac{1}{2}\langle \bar{1}11\rangle$, and red $b=\frac{1}{2}\langle 1\bar{1}1\rangle$. Continuous dislocations and loops evolve into a complex network where they pin to each other. a) Loop dipole b) dislocation pinned by loops c) loops decorating a dislocation d) complex junction. The loop radius is *r*_*l*_ = 5 nm.

**Figure 6:**
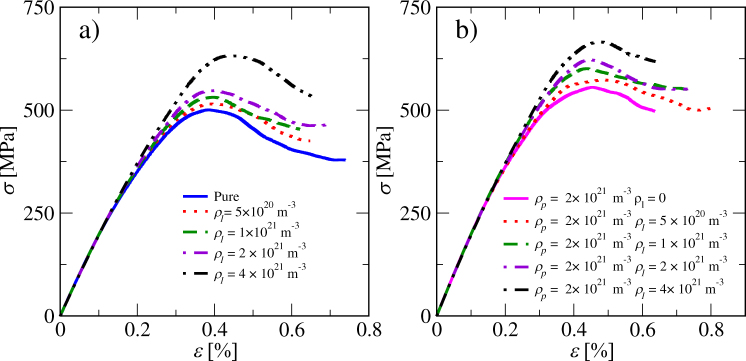
(**a**) Stress-strain curves of systems with only loops. Loop radius is *r*_*l*_ = 5 nm (**b**) Stress-strain curves with a fixed precipitate density and varying loop densities. Precipitate size parameter is *R*_*p*_ = 5 nm and the loop radius is *r*_*l*_ = 5 nm.

Similar loop-dislocation interactions were observed in our simulations. Generally the simulations with prismatic loops and straight dislocations progressed similarly to what is observed in the case of pure dislocation systems as well as in the dislocation-precipitate systems. The dislocations start to move with increasing stress and form junctions which serve as sources for dislocation multiplication generating a topologically complex network. Dislocation loops tend to move on their glide planes and form larger structures because of the mutual attraction of segments of different signs. This mechanism tends also to move the loops towards the continuous dislocations, resulting in loops decorating the line dislocations. Examples of these different mechanism are shown in Fig. 5. Stress strain-curves of systems with different densities of dislocation loops are plotted in Fig. 6a). They indicate that the yield stress increases as the loop density increases. Video of an example video is provided as Supplementary Material (Video 4).

**Figure 7:**
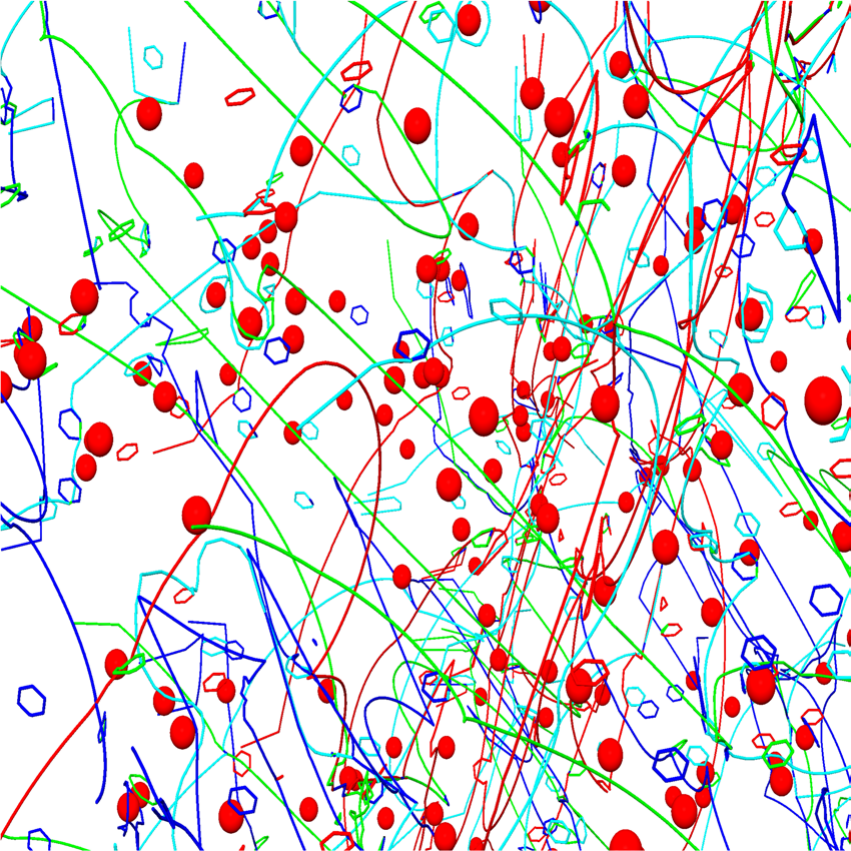
Close-up view of the 3D structure of a dislocation system with glissile dislocation loops and strong precipitates at a strain of *ε* = 0.74%. Different Burgers vectors are indicated with different colors as follows: dark blue $b=\frac{1}{2}\langle 111\rangle$, cyan $b=\frac{1}{2}\langle 11\bar{1}\rangle$, green $b=\frac{1}{2}\langle \bar{1}11\rangle$, and red $b=\frac{1}{2}\langle 1\bar{1}1\rangle$. Dislocations (lines) evolve into a complex network where they pin to each other. Overcoming the precipitates (red spheres) has left Orowan loops around some of the obstacles. Both loops and precipitates act as obstacles for dislocation motion. Precipitate size parameter is *R*_*p*_ = 5 nm and the loop radius is *r*_*l*_ = 5 nm.

When strong precipitates are added to the system, they tend to pin both line dislocations and loops as can be observed in Fig. 7. Straight dislocations bypass the precipitates with the Orowan mechanism, and Orowan loops are generated similarly to the case of the pure precipitate-dislocation systems. Examples of stress-strain curves of systems with a fixed precipitate density and varying number of dislocation loops are plotted in Fig. 6b). Video of an example simulation is provided as Supplementary Material (Video 5).

**Figure 8:**
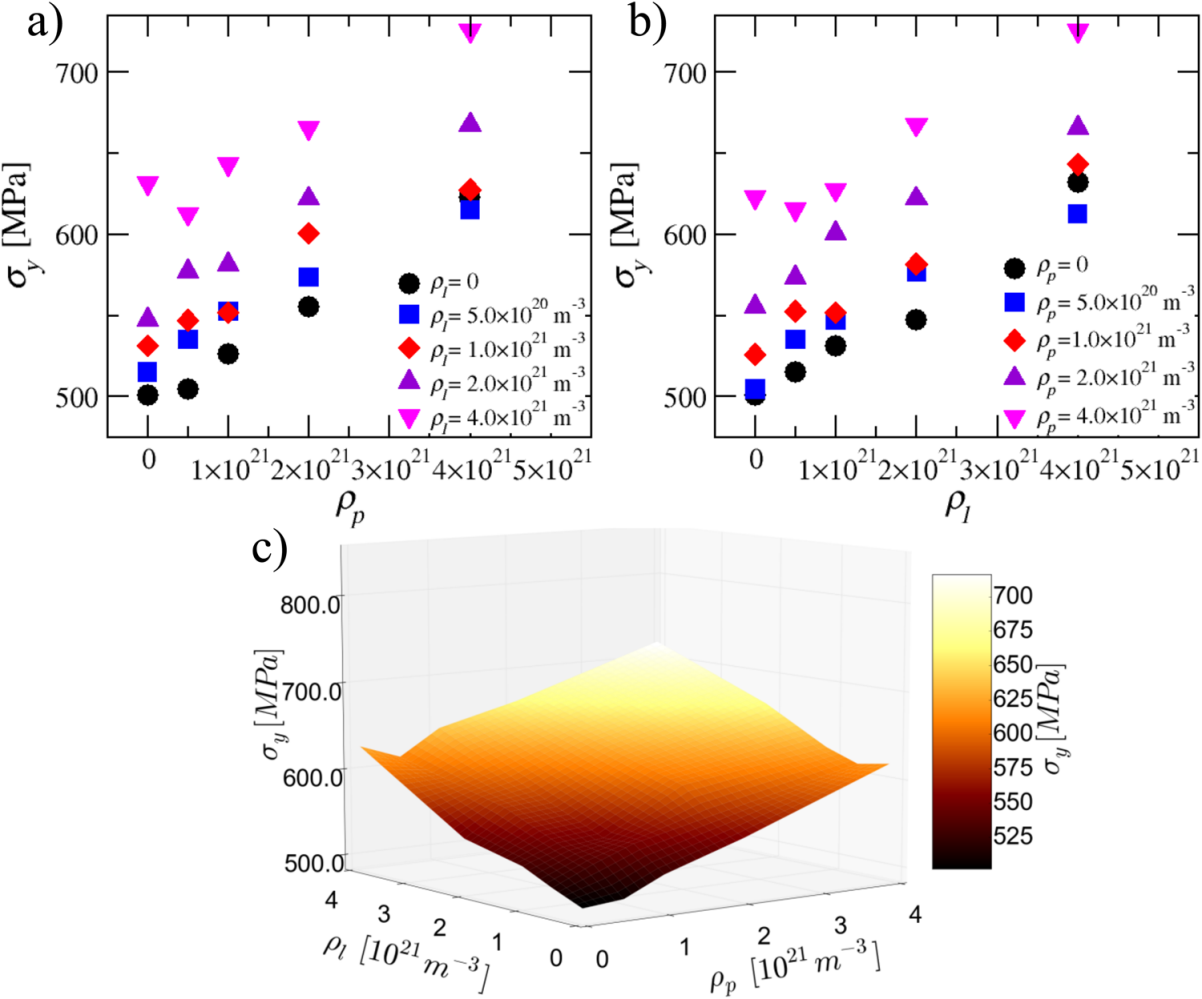
(**a**) Yield stress as a function of precipitate density for different loop densities. (**b**) Yield stress as a function of loop density for different precipitate densities. (**c**) A 3D surface plot of yield stress as a function of both loop and precipitate densities. Linear interpolation was used between the datapoints. Precipitate size parameter is *R*_*p*_ = 5 nm and the loop radius is *r*_*l*_ = 5 nm.

With the goal of comparing the relative contributions of dislocation loops and precipitates to the yield stress we performed simulations where the precipitate and loop densities were varied in the same range. The loop radius *r*_*l*_ and the precipitate size parameter *R*_*p*_ are both fixed to 5 nm. In Fig. 8 the yield stress is plotted as a function of the precipitate and dislocation loop densities, respectively. Yield stress increases as a function of the defect density in both cases. In the bottom part the same data is plotted as a surface in 3D. Linear interpolation is used between yield stress points to generate the surface. It can be observed that both types of obstacles contribute roughly equally to the yield stress.

## Discussion

We have simulated the effect of small strong precipitates and glissile dislocation loops on the yield stress of BCC iron in large-scale DDD simulations. Our simulations of the dislocation-precipitate system showed the basic properties of precipitate hardening in a multiscale framework. The presence of immovable precipitates leads to roughened dislocation lines and this is seen as increased dislocation density and strain hardening: yield stress of the crystal increases as a function of the precipitate number density. We compared the yield stress obtained from the simulations to an analytical expression for randomly positioned hard obstacles by Bacon *et al*.^48^ and found them approximately matching. The main reason for the differences is probably because of the quasistatic loading assumption used in obtaining the analytical expression, since our results have been obtained by considering rather high strain rates.

The reason for these high strain rates is that the mobility parameters obtained from MD are quite high when compared to typical ones used in previous DDD simulations^34^. Such a high mobility leads to a small timestep Δ*t* < 10^−14^ s in the simulations which forced us to consider a rather high strain rate $\dot{\varepsilon }={10}^{6}\,{{\rm{s}}}^{-1}$ to reach reasonable strains within accessible simulation times. This high strain rate introduces rate effects and consequently, the yield stress we found for a pure system is somewhat higher than experimentally observed for iron in the quasistatic limit.

Further complications arise from the fact that even though we use periodic boundary conditions, our simulation box of smallish size does not fully mimic bulk materials due to the size-effect “smaller is stronger”, i.e., a large system is weaker than a small system with a similar dislocation density even when using periodic boundary conditions^51^. Thus, while it is true that also the boundary conditions (open vs periodic) have an effect on the yield stress value, since the system sizes we consider are in the range of those of micropillars, these constitute a more relevant experimental reference system than bulk systems for stress scale comparisons. The main difference between our system and micropillars is that in the latter, dislocations can escape via the free surfaces which may lead to dislocation starvation in sufficiently small pillars. This is an important factor also in the case of irradiated systems, where SIA loops can be pushed out of the sample by the line dislocations. These processes are, however, expected to be more relevant at the later stages of plastic deformation, and less so at the initial yield point which is the main focus of our paper.

In the analysis of our simulation results for the yield stress, we used a linear rule of mixture for evaluating the stress contributions from precipitates and forest dislocations, respectively. Linear rule is a special case of the more general rule ${\sigma }_{y}={({\sigma }_{{\rm{pure}}}^{{\rm{k}}}+{\sigma }_{{\rm{precip}}}^{{\rm{k}}})}^{\frac{1}{k}}$. Based on their DDD simulations, Queyreau *et al*. suggested that the quadratic rule of mixture, k = 2.0, is more suitable for predicting flow stress of a dislocation-precipitate system when it is loaded so that a single slip system is active^28^. For our simulations we found that the linear rule was able to predict the yield stress better than the quadratic one. This seems reasonable because even if the precipitates were made impenetrable at their equator, the probability that a line dislocation meets a precipitate exactly at its equator is small. Thus, the precipitates form a system with effectively a few strong pinning points and many weaker ones. The difference to the result by Queyreau *et al*. can be caused by several reasons. First, we have included the pencil glide of screw segments which affects the strength of the dislocation-precipitate interaction. Secondly, we load the the system in tensile direction which introduces resolved shear stresses that move dislocations in multiple slip planes simultaneously, making collective dislocation motion more relevant. Thirdly, the quadratic result is obtained for the flow stress for high strain rates, while we looked at the stress for the initial yielding.

An interesting observation is that due to pencil glide, the screw segments can move on non-crystallographic planes, and thus the classical Orowan bypassing mechanism is altered in some instances. When a screw dislocation moves over a precipitate the trailing edge segments that it leaves around the precipitate can be in different parallel glide planes, and thus do not annihilate each other easily. Instead of a classical Orowan loop which lies on a plane, this generates a twisted noose around the precipitate, which stays connected to the parent line dislocation for some time. This bears similarity to experimental observations of screw dislocations in BCC materials where they unpin from obstacles by twisting around them, resulting in edge segments on different planes^52,53^. At long time scales and high temperatures the noose configuration may relax as the trailing edge segments can move via climb and annihilate each other^54^. The effect of this more complex dislocation-precipitate interaction on strain hardening is not easy to predict. Thus, comparative DDD simulations of precipitate hardening with and without pencil-glide motion taken into consideration are warranted.

In the present study the precipitate size was kept constant in contrast to real materials where the precipitate size follows a distribution. A topic for future work is the systematic study of the effect of different precipitate size distributions. Addition of Eshelby misfit stresses to the Gaussian potential would increase the realism of our precipitate implementation. Dislocation dynamics simulations based on the level set method have shown that dislocation can overcome misfit obstacles with several different mechanisms depending on the position where the dislocation cuts into the obstacle^55^. The effect of these mechanisms to the yield stress of large-scale multi-slip systems and the effects of cross-slipping and dislocation climb on *σ*_*c*_ are straightforward venues for future research.

The main results concerning systems containing both dislocation loops and precipitates was that even though loops interact with straight dislocations in a more complex manner than precipitates, i.e, they can be pushed around or absorbed by the line dislocations, their effect on the yield stress is of similar magnitude when they are approximately of similar size, and present in equal densities. In our simulations, which focused on capturing the initial yield point, there was no clear indication of the formation of loop-free channels or strain localisation. This is due to the fact that channel or shear band formation in crystals with SIA loops is typically observed for strains well beyond the yield point^34^. Hence, in our simulations where we focus on the dependence of the yield stress on the concentrations of loops and precipitates, we do not observe strain localization as our strains are not sufficiently large. Moreover, our simulations were performed in conditions in which the precipitates are frozen and not removed by dislocation flow. Precipitates can also get destroyed by plastic deformation, but the current model does not include a mechanism for that to happen, which would need an additional multiscale step in order to input the right rules from molecular dynamics. This implies that deformation band or channel formation in a system with a high density of strong precipitates cannot be expected as long as it is a consequence of the typical mechanism of debris removal by mobile dislocations. Similarly to the system with only line dislocations and dislocation loops, the channel formation in DDD simulations in any case takes place at plastic strains beyond the yield point^34^, and thus our observations about the yield strength would stand even if channel-inducing precipitate-dislocation interactions were added. Nevertheless, it would be an interesting (and computationally demanding) future research direction to try to reach higher strains such that, for instance, the possible deformation localization/channel formation could be addressed.

On the technical side these simulations showed that numerical load was decided by the precipitate size. The reason for this is that the resolution of the dislocation segment discretization has to be fine enough for the segments to “see” the precipitates. An improvement to the current implementation would be a re-meshing function that applies a finer discretization to those dislocation segments which are near precipitates. This would allow one to simulate systems with higher densities of dislocation loops and precipitates.

## Methods

### Discrete dislocation dynamics model

In 3D DDD simulations, dislocations are modeled by using a nodal discretization scheme: dislocation lines are represented by nodal points connected to their neighbors by straight segments. Changes in dislocation geometry are made possible by adding and removing these nodal points. The total stress acting on a node consists of the external part, resulting from the deformation of the whole crystal, and of the internal, anisotropic stress fields generated by the other dislocations within the crystal. The latter stress fields are computed by applying the well-known results of linear elasticity theory to the straight segments between nodes. Both of these fields generate forces which move the discretization nodes. The forces between dislocations themselves are divided to local and far-field ones. Forces between segments of nearby nodes and self-interaction of dislocations are calculated with explicit line integration of the Peach-Koehler force^39^. Far-field forces are calculated from the coarse-grained dislocation structure using a multipole expansion. Near the dislocation core, local interactions, such as junction formation, annihilation, etc., are introduced phenomenologically with input from smaller scale simulation methods (e.g., MD) and experimental results.

In this work we use a modified version of the open source DDD code ParaDiS^39^, where we have added a new obstacle data structure which is used to model immobile spherical defects like precipitates^38^. For simplicity, the obstacles interact with dislocation segments via a Gaussian potential $U(r)=A{e}^{-\frac{{r}^{2}}{{R}^{2}}}$. Thus, the interaction force between a dislocation segment and a precipitate is taken to be

4 $$F(r)=-\,\nabla U(r)=\frac{2Ar{e}^{-\frac{{r}^{2}}{{R}_{p}^{2}}}}{{R}_{p}^{2}},$$

where *A* is a force parameter which determines the strength of the dislocation-obstacle interaction, and *R*_*p*_ is a size parameter for the obstacle. The values of these parameters can be estimated from MD simulations^38^. The force field from the potential is continuous in space which is beneficial when considering the numerical stability of the simulation: there are no abrupt changes in the forces experienced by the discretization nodes which would lead to a decrease in the simulation time step. The Gaussian potential allows us to tune the defect strength from soft, shearable obstacles to strong impenetrable obstacles which the dislocations have to bypass via the Orowan mechanism. The Eshelby misfit stress-field is not included in this model so it is suitable for simulating local pinning points like incoherent precipitates. Further details of the obstacle implementation and multiscale framework can be found in ref.^38^.

### Data Availability

The datasets generated and analysed during the current study are available from the corresponding author on request.

## Electronic supplementary material

Video 1: Pure dislocation system

Video 2: Dislocation system with precipitates

Video 3: A loop is absorbed to a dislocation as a helical turn

Video 4: Dislocation system with loops

Video 5: Dislocation system with both precipitates and loops
